# The burden of adolescent motherhood and health consequences in Nepal

**DOI:** 10.1186/s12884-020-03013-8

**Published:** 2020-05-24

**Authors:** Rejina Gurung, Mats Målqvist, Zhou Hong, Pragya Gautam Poudel, Avinash K. Sunny, Srijana Sharma, Sangeeta Mishra, Nisso Nurova, Ashish KC

**Affiliations:** 1 Golden Community, Lalitpur, Nepal; 2 Department of Women’s and Children’s Health, Uppsala University, Uppsala, Sweden; 3 Department of Maternal and Child Health, Peking University Health Science Center, Beijing, China; 4 Department of Public Health, University of Tennessee, Knoxville, USA; 5 Ministry of Health and Population, Koshi Zonal Hospital, Koshi, Nepal; 6 Marie Stopes International, London, UK

**Keywords:** Adolescent mother, Adverse outcome, Preterm birth, Major malformation

## Abstract

**Background:**

Annually, 18 million babies are born to mothers 18 years or less. Two thirds of these births take place in South Asia and Sub-Saharan Africa. Due to social and biological factors, adolescent mothers have a higher risk of adverse birth outcomes. We conducted this study to assess the incidence, risk factors, maternal and neonatal health consequences among adolescent mothers.

**Methods:**

We conducted an observational study in 12 hospitals of Nepal for a period of 12 months. Patient medical record and semi-structured interviews were used to collect demographic information of mothers, intrapartum care and outcomes. The risks of adverse birth outcomes among adolescent compared to adult mothers were assessed using multivariate logistic regression.

**Results:**

During the study period, among the total 60,742 deliveries, 7.8% were adolescent mothers. Two third of the adolescent mothers were from disadvantaged ethnic groups, compared to half of adult mothers (66.1% vs 47.8%, *p*-value< 0.001). One third of the adolescent mothers did not have formal education, while one in nine adult mothers did not have formal education (32.6% vs 14.2%, *p*-value< 0.001). Compared to adult mothers, adolescent mothers had higher odds of experiencing prolonged labour (aOR-1.56, 95% CI, 1.17–2.10, p-0.003), preterm birth (aOR-1.40, 95% CI, 1.26–1.55, *p* < 0.001) and of having a baby being small for gestational age (aOR-1.38, 95% CI 1.25–1.52, *p* < 0.001). The odds of major malformation increased by more than two-fold in adolescent mothers compared to adult mothers (aOR-2.66, 95% CI 1.12–6.33, p-0.027).

**Conclusion:**

Women from disadvantaged ethnic group have higher risk of being pregnant during adolescent age. Adolescent mothers were more likely to have prolonged labour, a preterm birth, small for gestational age baby and major congenital malformation. Special attention to this high-risk group during pregnancy, labour and delivery is critical.

## Background

In the Sustainable Development Goal era, the planet has witnessed the largest adolescent (10–19 years) cohort population in human history. There are approximately 1.2 billion adolescents worldwide, with more than 85% living in low- and middle-income countries [1]. The adolescent population has the potential to transform societies if provided with an enabling and empowering environment for a healthy life [2, 3]. Adolescence is a period of rapid brain development, alongside social, emotional and cognitive changes that prepare adolescents to transition to adulthood [4, 5]. The Lancet Commission for Adolescent Health and Wellbeing highlights the opportunities and challenges in improving adolescent health in the SDG era [6, 7]. Among the key challenges for better health of adolescents is the need to address adolescent pregnancy and health outcomes of mother and fetus.

In 2013, globally, 16 million girls between the ages of 15–19, and two million girls under age 15, became pregnant [8]. There has however been progress over the past 60 years. In the poorest regions of the world, birth rates in 1950–55 averaged 170 births per 1000 among girls aged 15–19. In 2010 this was decreased to 106 per 1000 [9]. However, this birth rate is still four times higher than in the high income regions of the world [9].

According to the United Nations Population Fund (UNFPA), one third of all women between the ages 20 and 24 years report being married during adolescent period [10, 11]. Globally and in Nepal, adolescent contraceptive prevalence rate is lower, and unmet need for contraception is greater for adolescents thank any other age group [12]. Contraception is one of the most effective ways to prevent pregnancy and in effect reduce adverse outcomes amongst both adolescents and babies. Even though adolescent pregnancy is a global problem, adolescents and young adults have been left behind in global health and social policy [13]. Given, the attention required for improving health and development of adolescent, in September 2015, the UN Secretary-General’s Global Strategy for Women’s, Children’s and Adolescents’ Health provided an investment opportunity in adolescent health and wellbeing [14, 15].

Pregnancy during the adolescent period entails considerable risk for complications during labour such as placental tear, obstruction and long-term consequences of obstetric fistulae [16]. Adolescent girls who become pregnant are more likely to be from a lower economic status than their peer, with poorer nutrition and general health status. This in turn increases the likelihood of fetal, perinatal and maternal death and disability [17]. Further, an unmarried pregnant adolescent is likely to face social discrimination and more risk of violence, as pregnancy out of wedlock can a social stigma in low- and middle-income settings [18].

In Nepal in 2016, the median age at first marriage is 17.9 years among women. 52% of women are married by age 18, as compared to 19% of men [19]. The adolescent fertility rate has reduced from 110 per 1000 women in 2000 to 60.5 per 1000 women in 2017 [20]. The Nepal Health Sector Strategy 2016–2021 aims to reduce the adolescent fertility rate from 2.3 to 2.1 by 2021 [21]. However, the rate of progress seems to be slow [22]. There has been global and national level advocacy to reduce the rate of adolescent pregnancy and yet in 2017, 120,000 adolescent were pregnant in Nepal [23]. We conducted this study to assess the incidence, risk factors, maternal and neonatal consequences among adolescent mothers in Nepal.

## Method

The study has been reported as per the checklist for STrengthening the Reporting of OBservational studies in Epidemiology (STROBE) [24].

### Design and setting

This is an observational study conducted for a period of 12 months in 12 public hospitals of Nepal between July 2017–June 2018 nested within a large-scale neonatal resuscitation program [25, 26]. These 12 public hospitals range from primary referral hospital to secondary referral hospitals located across the country. The volume of annual delivery in each hospital ranged from 1000 to 12,000 per year (Additional file 1).

All of these hospitals provided Comprehensive Emergency Obstetric and Neonatal Care Services [27]. All the hospitals provided family planning, postnatal care and immunization services.

### Eligibility criteria

All women admitted in the hospitals for delivery with gestational age 22 week or more were eligible to be enrolled in the study. Women consented to be enrolled in the main study at the time of admission for delivery. Written consent was taken from the women. Women who gave birth outside these hospitals but were admitted for postnatal complication care were excluded from the study.

### Data collection and management

To collect maternal and newborn health data, a surveillance system was established in all the hospitals, with data collectors collecting clinical information of mothers and newborns using a data retrieval form (Additional file 2). In addition, a semi-structured interview was conducted with mothers at the time of discharge to gather information regarding socio-demographic characteristics and antenatal care (Additional file 3). Following the data extraction and interviews, the completed forms were assessed by a data coordinator on each site. Additionally, the data coordinator indexed the information sheets at the end of each day. Every week, the completed forms were sealed in an opaque envelope and sent to the main research office in Kathmandu. Forms were then quality checked for completeness. Data cleaning was performed in Census and Survey Processing System (CSPro) every month. The cleaned data were exported in Statistical Package for the Social Sciences (SPSS) for data analysis. The exported data were stored in a secured external hard drive to ensure the privacy and safety of the data. Personal information was removed before performing any data analysis. All hard copies of the information sheet were indexed and stored in a secured room at the research office for future references as per the ethical guidelines.

#### Variables included in the study

##### Socio-demographic and obstetric characteristics

*Education* level in mother was categorized as formal education or not.

*Ethnicity of mother* was categorized as Dalit, Janjati, Madhesi, Muslim, Chettri/Brahmin, and other castes based on hierarchical caste system of Nepal [28]. Ethnicity was categorized as disadvantageous group (dalit, janjati and muslim) and relatively advantageous group (Madhesi, Chettri/Brahmin and other castes) based on hierarchical caste system of Nepal.

*Severe anemia:* the serum hemoglobin 7 g per deciliter or less at admission.

*Antepartum hemorrhage:* vaginal bleeding before onset of labor [29].

*Hypertensive disorder*: classified by maternal diastolic blood pressure greater than or equal to 90 mmHg in two separate recordings [29].

*Prolonged labor:* cervical dilation that does not move beyond 4 cm after 8 hrs of regular contractions, or cervical dilation lying to the right of the alert line on the partogram [29].

*Preterm birth:* birth of the baby with gestational age less than 37 weeks. The variable categorized as less than 37 week and 37 weeks or more.

*Small weight for gestational age (SGA)*: birth weight less than 10 centile as per the gestational age [30]. The variable categorized as small weight for gestational age and appropriate weight for gestational age (10 centile or more).

*Major malformation*: major congenital malformation-malformation of neural tube defect, cardiac and gastro-intestinal.

*Antepartum stillbirth*: delivery of any fetus occurring after 22 weeks of gestation or with a birth weight more than 500 g that had with no FHS at admission and no signs of life at birth.

*Intrapartum stillbirth:* delivery of a fetus occurring after 22 weeks’ gestation or with a birth weight 500 g or more, who had FHS at admission and no signs of life at birth.

*Pre-discharge neonatal mortality:* neonatal death before discharge from the hospital.

#### Statistical analysis

Differences in the demographic, obstetric and neonatal characteristics between the adolescent and adult mother was assessed using the Pearson’s chi-squared test (χ2). Variables which had a difference of *p* < 0.20, was included in a multi-variate logistic regression analysis. During data analysis, missing values were treated as missing. All the statistical analyses were performed using SPSS version 25.

## Results

**Fig. 1:**
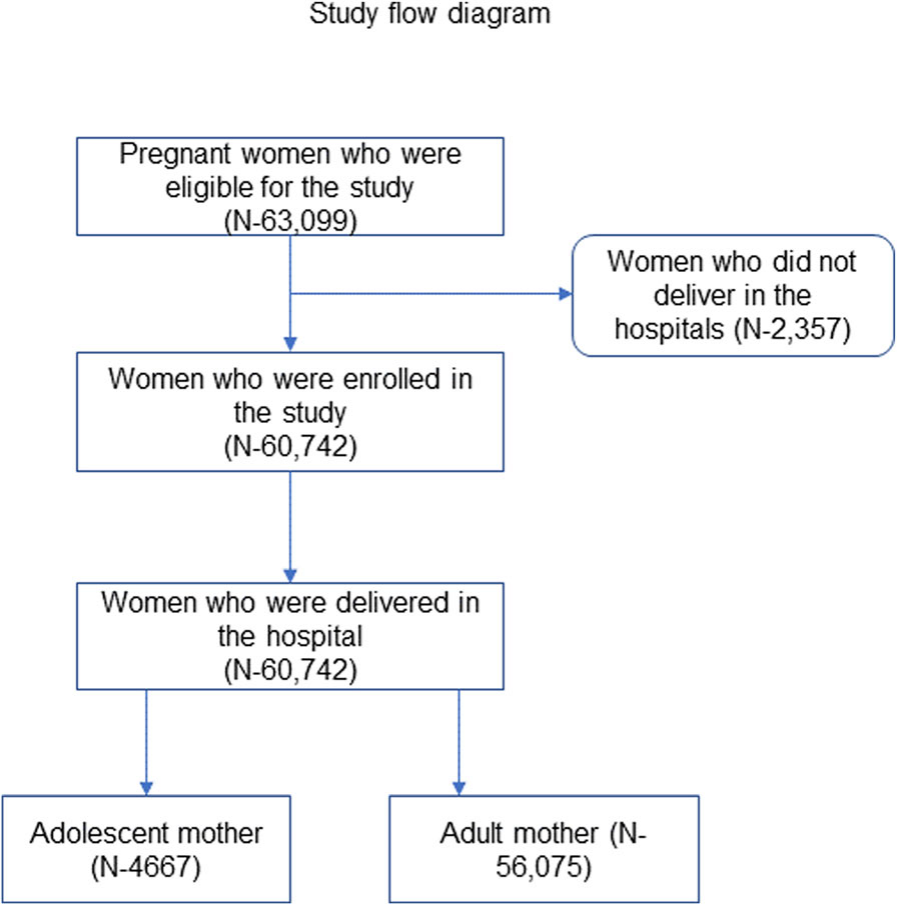
Study flow diagram

A total of 63,099 pregnant women admitted in the participating hospitals were eligible for study inclusion. Among these, 60,742 women delivered at the respective hospital and were enrolled. Among the enrolled women, 7.8% were adolescents (Fig. 1). There was a higher proportion of women from a disadvantaged ethnic group among adolescents compared to adult women (66.1% vs 47.8%, *p*-value < 0.001). More adolescent mothers lacked formal education compared to adult mothers (32.6% vs 14.2%, *p*-value< 0.001). (Table 1).

**Table 1:** Demographic, obstetrics and neonatal characteristics between adolescent and adult mothers

	Adolescent mother (N-4667)	Adult mother (N-56075)	Total population (N-60742)	*p*-value
Disadvantaged ethnic group	3083 (66.1%)	26,830 (47.8%)	29,913 (49.2%)	*p* < 0.001
No formal education	1263 (32.6%)	6610 (14.2%)	7873 (15.6%)	*p* < 0.001
Severe anemia	13 (0.3%)	186 (0.3%)	199 (0.3%)	0.542
Preeclampsia	46 (1.0%)	538 (1.0%)	584 (1.0%)	0.778
APH	14 (0.3%)	195 (0.4%)	209 (0.4%)	0.636
Prolonged labour	62 (1.3%)	469 (0.8%)	531 (0.9%)	0.001
Small for gestational age	764 (16.4%)	6473 (11.5%)	7237 (11.9%)	< 0.001
preterm	659 (14.1%)	5305 (9.5%)	5964 (9.8%)	< 0.001
Major malformation	9 (0.2%)	38 (0.1%)	47 (0.1%)	0.009
Antepartum stillbirth	18 (0.4%)	225 (0.4%)	243 (0.4%)	0.88
Intrapartum stillbirth	45 (1.0%)	392 (0.7%)	437 (0.7%)	0.039
Pre-discharge mortality	90 (2.0%)	829 (1.5%)	919 (1.5%)	0.015

Results indicate that there was higher proportion of poor medical complications and conditions among the adolescent mothers. Adolescents had more prolonged labour compared to adult mothers (1.3% vs 0.8%, *p*-value = 0.001), the prevalence of babies born with small weight for gestational age was higher among the adolescent mothers compared to adult mothers (16.4% vs 11.5%, *p*-value < 0.001), and the incidence of preterm birth was higher among adolescent mothers compared to adult mothers (14.1% vs 9.5%, *p*-value< 0.001). The share of babies born with major malformation was higher from mother who were adolescent than adult (0.2% vs 0.1%, *p* = 0.009). Adolescent mothers also had higher intrapartum stillbirth rate than adult mothers (10.0 per 1000 birth vs 7.0 per 1000 birth, *p*-value = 0.039). Pre-discharge neonatal mortality was higher among adolescent mother compared to among adult mothers (2.0% vs 1.5%, *p*-value = 0.015) (Table 1).

**Fig. 2:**
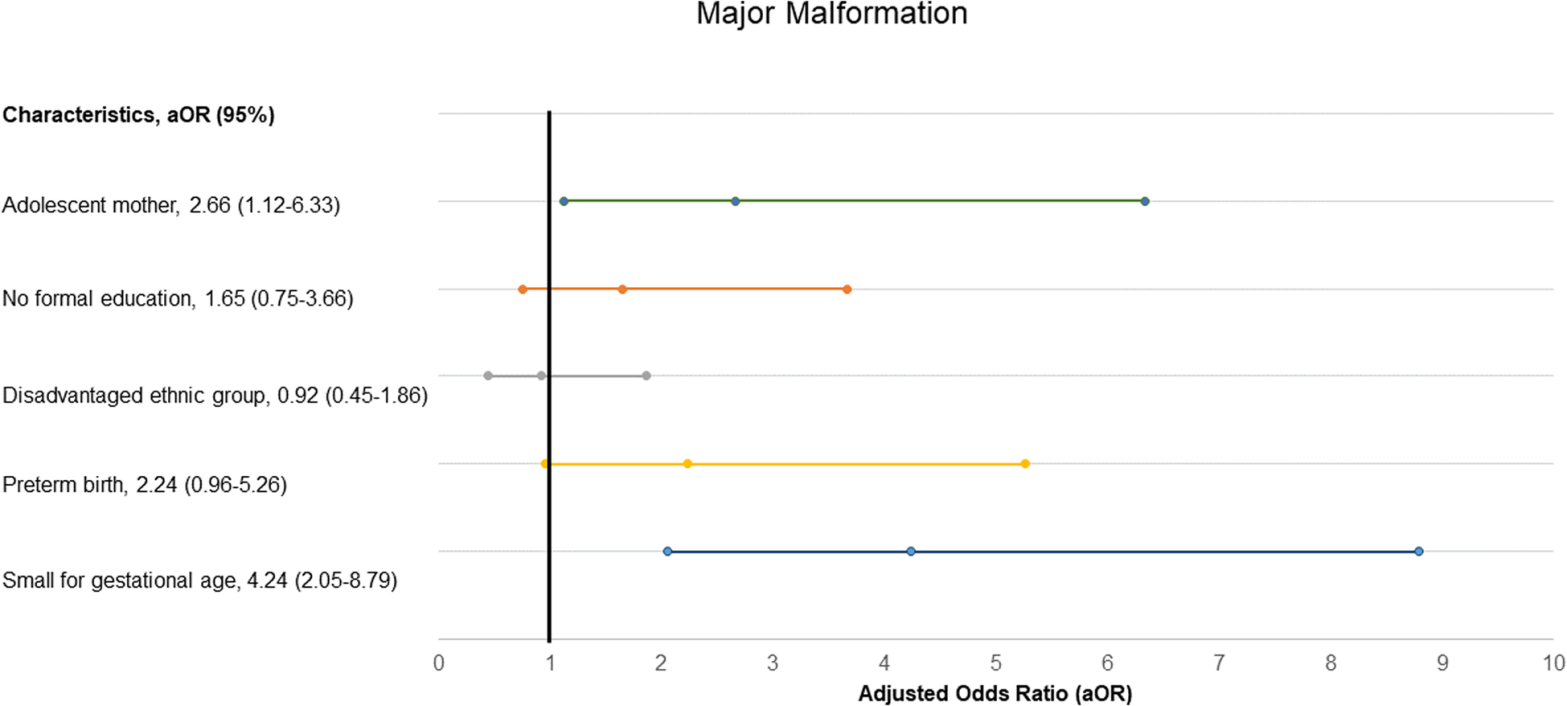
Level of association of adolescent motherhood with major malformation

**Fig. 3:**
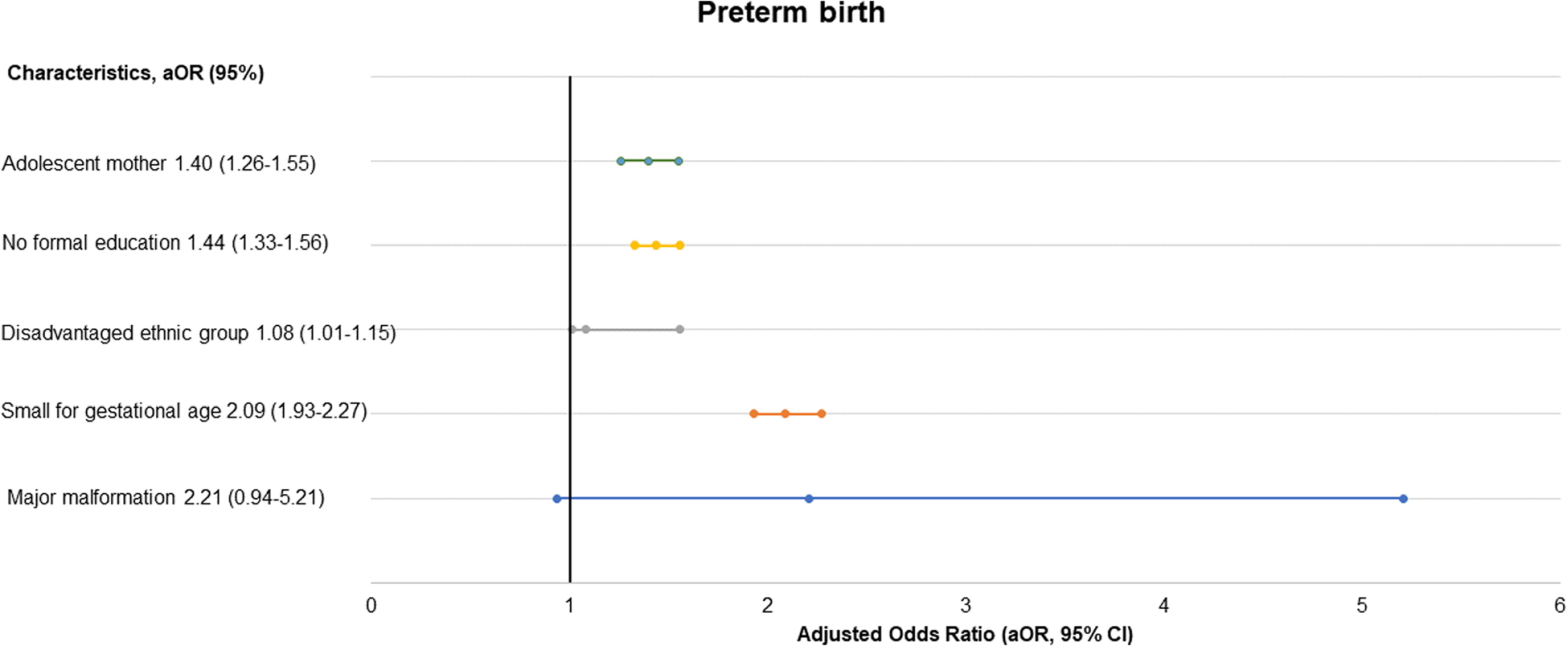
Level of association of adolescent motherhood with preterm birth

**Fig. 4:**
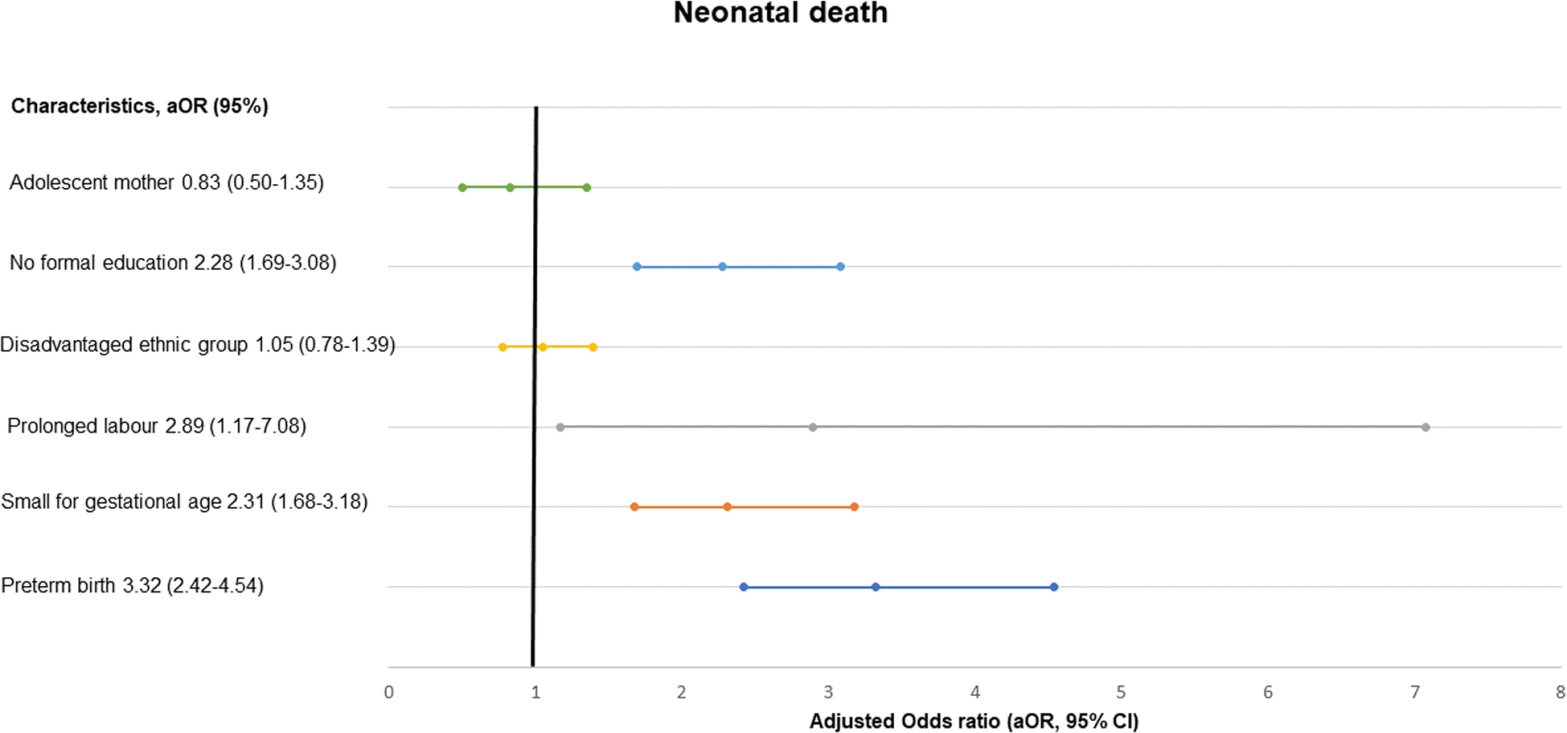
Level of association of adolescent motherhood with neonatal death

In the multi-variate analysis, adolescent mothers had a 56% higher likelihood for prolonged labour than adult mothers (aOR- 1.56, 95% CI, 1.17–2.10, *p*-value = 0.003) (Table 2). Adolescent mothers had a 38% higher likelihood of having a baby being small weight for gestational age than adult mothers (aOR-1.38, 95% CI, 1.25–1.52, *p*-value< 0.001) (Table 3), and an almost three-fold risk to have major malformation (aOR-2.66, 95% CI, 1.12–6.33, *p*-value-0.027) (Fig. 2). Adolescent mother had 40% higher likelihood of preterm birth compared to adult mothers (aOR-1.40, 95% CI, 1.26–1.55, *p*-value < 0.001) (Fig. 3). The adolescent mothers had no significant association with pre-discharge mortality (aOR-0.83, 95% CI, 0.50–1.35, *p*-value = 0.44) (Fig. 4).

**Table 2:** Risk of prolonged labour in adolescent mothers in a multi-variate analysis

	Prolonged labour	
	aOR (95% CI)	*p*-value
*Adolescent mother*	*1.56 (1.17–2.10)*	*0.003*
No formal education	0.72 (0.54–0.95)	0.02
Disadvantaged ethnic group	1.57 (1.30–1.90)	< 0.001
Small for gestational age	1.27 (0.96–2.67)	0.093
Major malformation	3.33 (0.45–24.7)	0.238
Preterm birth	0.63 (0.42–0.93)	0.021

**Table 3:** Risk of small for gestational age in adolescent mother in comparison with adult mother in multi-variate analysis

	Small for Gestational age	
	aOR (95% CI)	*p*-value
*Adolescent mother*	*1.38 (1.25–1.52)*	*< 0.001*
No formal education	1.23 (1.14–1.33)	< 0.001
Disadvantaged ethnic group	0.92 (0.87–0.98)	0.005
Major malformation	4.24 (2.05–8.78)	< 0.001
Preterm birth	2.08 (1.92–2.26)	< 0.001

## Discussion

Results from this study indicate that women from disadvantaged ethnic group have a higher likelihood of being an adolescent mother compared to the advantaged ethnic group. Pregnant adolescents are at a higher risk of adverse birth outcomes compared to adult women being more likely to experience prolonged labour, preterm birth and having a baby that is small for gestational age. There was also an increased risk of having a baby with a major malformation among the adolescent mothers compared to adult mothers. Our results could however not detect any increased risk of pre-discharge mortality among babies born to adolescent mothers.

In Nepal, caste and ethnicity has been the center-piece of the social hierarchy [28]. Families from higher caste or relatively advantaged ethnic group are more likely to have access to education and wealth than in the lower caste or disadvantaged ethnic group. This access may be a preventative factor for early marriage and adolescent pregnancy in the advantaged ethnic group. Furthermore, gender discrimination is relatively higher among the disadvantaged ethnic group [31] . A longitudinal study aimed to assess the factors for incident unwanted and unplanned pregnancies among adolescent women in South Africa showed that higher socioeconomic status was protective for both unplanned and unwanted pregnancies (OR 0.69; 95% CI 0.58–0.83 and OR 0.78; 95% CI 0.64–0.96) [32].

Adolescent girls who do not access education are also deprived of sexual education and information on the benefits to pregnancy, as well as the health consequences of early pregnancy [7]. Girls who are better educated are shown to have greater decision-making power in relation to accessing and navigating health service [7]. Furthermore, adolescent girls in Nepal are more likely to have an adolescent pregnancy if they do not access education [31]. A cross-sectional study conducted among 457 women, age between 14 and 24 years carried out in Rupandehi district of Nepal showed that lack of education for the girls was the key contributing factor for adolescent pregnancy [33].

Adolescent pregnancy has been associated with obstetric and neonatal complication. A retrospective review of 15,498 pregnant women in South India has shown that girls aged ≤19 had higher incidence of anemia, low birth weight, and a significantly lower incidence of caesarean sections/perineal tears in comparison to adult mothers [34].

Preventing first pregnancy and subsequent pregnancy among adolescent mother is important to reduce the morbidity and mortality [32]. Subsequent pregnancy among adolescent mother is a major risk that has implication for woman’s life [32]. Therefore, strategies to prevent first and subsequent pregnancies among adolescent women is critical. Education has become a major strategy in addressing adolescent health problem and it is worth investing to increase the extent and quality of schooling [35]. Investment in health and education not only play a critical role in shaping the lives of adolescents in resource-poor settings, but it also has added benefits for generating high economic and social returns [36]. Therefore, investing in education for girls in Nepal has broader ranging benefits for society but can also serve as an avenue to prevent adolescent pregnancies.

### Methodological consideration

The study conducted rigorous surveillance to assess the various demographic and obstetric factors among the adolescent mothers. One of the major strengths of this study is inclusion of a large sample from 12 public hospitals located in different geographical locations in Nepal. Further, another strength of this study is inclusion of all in-born babies, including antepartum still births.

### Limitations

There are several limitations in the study. First, we conducted an interview at the time of discharge to gather information regarding socio-demographic characteristics and the quality of care. There might be interviewer’s bias while gathering information. Second, we also could not interview mothers of still birth which might have resulted in missing information. Third, though prior studies have shown association between pre-eclampsia and adolescent births, we could not explore pre-eclampsia as it might have been under-reported.

## Conclusion

Reducing adolescent motherhood will require investment in education of girls and access to information to prevent pregnancy. Because adolescent pregnancy is associated with poor obstetric and neonatal outcome, improving quality of intrapartum care for high-risk mothers can reduce risk of poor outcomes. In Nepal, health facilities need to provide special attention and care for these high-risk mothers, to address the health needs of adolescent girls and prevent long-term morbidities and mortality.

## Supplementary information

**Additional file 1.** Estimated deliveries at the selected hospital (year:2015)

**Additional file 2.** NePeriQIP registry form

**Additional file 3.** NePeriQIP Client Exit Interview form

## Data Availability

The datasets generated and/or analysed during the current study are available in the google drive repository https://drive.google.com/drive/folders/17NOYhGln1hEUyE6_ihbzowSVQwnDH0hb

## Abbreviations

aOR: Adjusted odds ratio
UNFPA: United Nations Population Fund
STROBE: STrengthening the Reporting of OBservational studies in Epidemiology
CSPro: Census and Survey Processing System
SPSS: Statistical Package for Social Sciences
SGA: Small weight for gestational age
FHS: Fetal heart sound
