# Supplementary material for: The burden of adolescent motherhood and health consequences in Nepal
Source: BMC Pregnancy Childbirth. 2020 May 24;20:318. doi: 10.1186/s12884-020-03013-8 (PMC7245914; doi:10.1186/s12884-020-03013-8)
Supplement: Supplementary file 1 — Additional file 1. Estimated deliveries at the selected hospital (year:2015) [file 12884_2020_3013_MOESM1_ESM.docx]

Appendix 1 Estimated deliveries at the selected hospital (year:2015)

| **Name of hospital** | **Total deliveries per year** | **Intrapartum still births** | **Neonatal mortality** |
| --- | --- | --- | --- |
| Western Regional Hospital | 7382 | 87 | 25 |
| Mid-Western Regional Hospital | 4184 | 21 | 118 |
| Bardiya District Hospital | 1033 | 3 | 19 |
| Bharatpur Hospital | 11086 | 80 | 52 |
| Seti Zonal Hospital | 8009 | 2 | 236 |
| Nuwakot District Hospital | 1683 | 11 | 16 |
| Koshi Zonal Hospital | 6048 | 56 | 36 |
| Rapti Sub-Regional Hospital | 4216 | 43 | 29 |
| Nawalparasi District Hospital | 856 | 5 | 5 |
| Lumbini Zonal Hospital | 8746 | 97 | 125 |
| Bheri Zonal Hospital | 5636 | 7 | 221 |
| Pyuthan District Hospital | 1863 | 25 | 37 |
