# Supplementary material for: The burden of adolescent motherhood and health consequences in Nepal
Source: BMC Pregnancy Childbirth. 2020 May 24;20:318. doi: 10.1186/s12884-020-03013-8 (PMC7245914; doi:10.1186/s12884-020-03013-8)
Supplement: Supplementary file 2 — Additional file 2. NePeriQIP registry form [file 12884_2020_3013_MOESM2_ESM.pdf]

ID NUMBER: ______________________

Form 1

NePeriQIP Registry Data

Data Collector	Name……………………………………….………………………<br>Code………………………………………………………………..

Data ID	Information		Write or circle where applicable	Notes
**PART A: BACKGROUND INFORMATION**				
101.	Mother's first name			
102.	Mothers' last name			
103.	Inpatient number			
104.	Age of mother (completed years)		☐☐	
105.	Caste			
106.	Ethnicity code			
107.	Address	District		
		Municipality		
		Ward		
108.	Mobile		☐☐☐☐☐☐☐☐☐☐	
109.	Parity		Nullipara (never carried a pregnancy >22 wks)……1	
			Primipara (1 birth)…………....……………………..…..2	
			Multipara (2-5 births)..…………………......................3	
			Grand multipara (>5 births)…………......................4	

	Date (BS) dd/mm/yyyy	Signature
Form completed:		
Data entered into database:		

PART B: PRE-DELIVERY DETAILS					
**Part B1: Complications during pregnancy (based on ANC records)**					
110.	**Complications recorded**	***Yes***	**No**	***NR***	
110a.	*Vaginal bleeding*	1	0	98	
110b.	*Premature rupture of membranes (PROM)*	1	0	98	
110c.	*Abdominal pain*	1	0	98	
110d.	*Decreased foetal movement*	1	0	98	
110e.	*No foetal movement*	1	0	98	
110f.	*Muscle or joint pain*	1	0	98	
110g.	*Lethargy*	1	0	98	
110h.	*Convulsion*	1	0	98	
110i.	*Coma/unconscious*	1	0	98	
110j.	*Disturbed vision*	1	0	98	
110k.	*Oedema*	1	0	98	
110l.	*Headache*	1	0	98	
110m.	*Hypertension during pregnancy (≥140/90 mmHg)*	1	0	98	
110n.	*Pre-existing hypertension*	1	0	98	
110o.	*Glucose in urine*	1	0	98	
110p.	*Protein in urine*	1	0	98	
110q.	*Severe anaemia (Hb <7mg/dL)*	1	0	98	
110r.	*Frequent micturation*	1	0	98	
110s.	*Others (specify)*				
111.	Place making referral				
112.	Recent FHS recorded	Yes, normal (110-160 BPM)……………..………........1<br>Yes, abnormal (<110 BPM or >160 BPM)……2<br>Absent…………………………………………..…....3<br>Not recorded……………………………………....98			

Part B2: Condition at the time of admission					
113. **Provisional diagnosis of any complication recorded at the time of admission**		***Yes***	***No***	***NR***	
113a.	*Antepartum haemorrhage (APH)*<br>*Abruptio placenta/ruptured uterus/placenta previa*	1	0	98	
113b.	*Postpartum haemorrhage (PPH)*<br>*Atonic/tear/retained placenta/inverted uterus/ruptured uterus*	1	0	98	
113c.	*Prolonged labour (>12 hours active phase)*	1	0	98	
113d.	*Hypertensive disorder (Chronic Hypertension/Pregnancy Induced Hypertension/Pre-Eclampsia/Eclampsia)*	1	0	98	
113e.	*Malpresentation*	1	0	98	
113f.	*Malposition*	1	0	98	
113g.	*Prolapsed cord*	1	0	98	
113h.	*Chorioamnionitis*	1	0	98	
113i.	*Premature rupture of membrane (PROM)*	1	0	98	
113j.	*Pre-term labour*	1	0	98	
113k.	*Foetal distress in labour*	1	0	98	
113l.	*Foetal death*	1	0	98	
113m.	*Decreased foetal movement*	1	0	98	
113n.	*Foetal congenital anomaly*	1	0	98	
113o.	*Abdominal pain*	1	0	98	
113p.	*PV leaking*	1	0	98	
113q.	*Other (specify):*				
114.	Foetal heart rate at admission	Yes, normal (110-160 BPM)………………………….....1<br>Yes, abnormal (<110 BPM or >160 BPM)…………...2<br>Absent…..…………………………..…………....3<br>Not recorded………..……………................98			
115.	If foetal heart rate recorded	Date (BS) dd/mm/yyyy	☐☐☐☐☐☐☐☐		
		Time (hh:mm) 24-hr	☐☐☐☐		
116.	Stage of labour	Not in labour……..…………..………………..1<br>Latent stage of active labour……. ....2<br>First stage of active labour.............................3<br>Second stage of labour… ....4<br>Third stage of labour……....… 5			

PART C: DELIVERY DETAILS			
117.	Partograph use	Yes, completely filled……………….1<br>Yes, partially filled……………….….2<br>Not filled………………………….…..98	
118.	Foetal heart rate monitoring recorded during delivery	Yes, as per protocol….……….……..….1<br>Yes, sporadically (> once)……………..2<br>Yes, only once………….…..…………...3<br>Not recorded…………………..…......98	
119.	Induction of labour	Induction with prostaglandins…..…..1<br>Induction with amniotomy………….....2<br>Induction with oxytocin……………..3<br>No………………………………………..0<br>Not recorded……………….…..…. 98	
120.	Received prophylactic antibiotics	Yes………………………………….1<br>No…………………………………….0<br>Not recorded…………………..98	
121.	Mode of delivery	Spontaneous vaginal………………......1<br>*If vaginal delivery, go to 122*<br><br>Instrumental……………..…….…..... 2<br>*If Instrumental delivery, go to 121*<br><br>Assisted breech delivery……….......3<br>*Assisted Breech Delivery, go to 122*<br><br>Emergency CS………………….…..4<br>Elective CS………….……….........5	If Emergency or Elective CS, go to 125
122.	Reason for CS (multiple response)	Prolonged labour………..…....…..1<br>Foetal distress……………………......2<br>Abnormal lie………………………......3<br>Obstetric haemorrhage…..…….....4<br>Multiple pregnancy……….…....…...5<br>Previous scar……………..…….…....6<br>Maternal request……………….....7<br>Other (specify)………..……...….....	
123.	Reason for instrumental delivery	Prolonged labour…………………...1<br>Foetal distress……..……………….…...2<br>Other (specify)…..…..………….......	
124.	Mother given Oxytocin (for augmentation of labour)	Yes……………………………….....1<br>No……………………………….......0<br>Not recorded……………………...98	
125.	Multiple delivery	Yes………………………………….....1<br>No…………………………………......0	

PART D: NEWBORN DETAILS					
126.	Date of delivery (BS) dd/mm/yyyy	☐☐☐☐☐☐☐☐			
127.	Gestational age by LMP (weeks)	☐☐ + ☐<br>Not known………………… 0<br>Not recorded……………..98			
128.	Gestational age by ultrasound (weeks)	☐☐ + ☐<br>Not recorded……………..98			
		Single or Twin 1		Twin 2	
129.	Time of delivery (hh:mm)	☐☐☐☐		☐☐☐☐	
130.	Sex of baby	Girl…………………1<br>Boy………………..0		Girl…………………1<br>Boy………………..0	
131.	Birth weight (grams)	☐☐☐☐		☐☐☐☐	
132.	Delivery outcome<br>***(If Stillbirth, go to 150)***	Live birth…………..1<br>Stillbirth……………2		Live birth…………...1<br>Stillbirth……………2	
133.	APGAR at 1 minute	☐☐		☐☐	
134.	APGAR at 5 minutes	☐☐		☐☐	
135.	Malformation recorded	Yes………………..1<br>No………………….0		Yes……………….…1<br>No………………….0	
136.	Newborn given vitamin K	Yes…………………....1<br>Not recorded………0		Yes…………………....1<br>Not recorded…...… 0	
137.	Newborn body temperature recorded	Yes……………….…1<br>No………………….0		Yes………………… 1<br>No………………..0	
138.	Respiratory rate recorded	Yes………………..1<br>No………………….0		Yes………………..1<br>No………………….0	
139.	Medical examination of baby recorded	Yes………………..1<br>No………………….0		Yes………………..1<br>No………………….0	
140.	Neonatal morbidity recorded<br>***(If No, go to 143)***	Yes………………..1<br>No………………….0		Yes………………..1<br>No………………….0	
141.	Type of morbidity<br>(multiple response)	Yes	No	Yes	No
	a. Birth asphyxia	1	0	1	0
	b. Neonatal encephalopathy	1	0	1	0
	c. Hyper-bilirubinaemia	1	0	1	0
	d. Meconium aspiration	1	0	1	0
	e. Neonatal sepsis	1	0	1	0
	f. Respiratory distress syndrome	1	0	1	0
	g. Hypoglycaemia	1	0	1	0
	h. Retinopathy of prematurity	1	0	1	0
	i. Others	1	0	1	0

142.	If other cause of morbidity, please specify		
143.	Treated for sepsis	Yes…………………1<br>Not recorded …….0	Yes………………..1<br>Not recorded ……0
144.	Newborn transferred to other facility ***(If Not recorded, go to 146)***	Yes…………………1<br>Not recorded …….0	Yes…………………1<br>Not recorded ……..0
145.	Day of facility transfer (BS)		
146.	Newborn transferred to another ward (***If No, go to 148)***	Yes, SNCU/NICU……1<br>Yes, pediatric ward…2<br>No…………………….0	Yes, SNCU/NICU……1<br>Yes, pediatric ward…2<br>No…………………….0
147.	Day of transfer to ward (BS)		
148.	Newborns survived first 24 hours ***(If No, go to 151)***	Yes…………………1<br>No……………….. 0<br>Not known……….9	Yes…………………1<br>No………………..0<br>Not known……….9
149.	Newborns survived first 7 days ***(If No, go to 151)***	Yes…………………1<br>No……………….. 0<br>Not known……….9	Yes…………………1<br>No……………….. 0<br>Not known……….9
150.	Newborns alive at discharge ***(If Yes, got ot 157)***	Yes…………………1<br>No………………..0<br>Not known……….9	Yes…………………1<br>No……………….. 0<br>Not known……….9
151.	Date of death (BS) dd/mm/yyyy		
152.	Time of death (hh:mm)		
153.	Perinatal death audit completed ***(If No, go to 155)***	Yes…………………1<br>No…………………0	Yes…………………1<br>No…………………0
154.	If yes, was it completed within 24 hours?	Yes…………………1<br>No…………………0<br>Time not recorded…2	Yes…………………1<br>No…………………0<br>Time not recorded….2

155.	Cause of death according to journal	Yes	No	Yes	No
	Congenital abnormalities	1	0	1	0
	Preterm birth	1	0	1	0
	Birth asphyxia	1	0	1	0
	Sepsis/pneumonia	1	0	1	0
	Intrapartum stillbirth	1	0	1	0
	Antepartum stillbirth	1	0	1	0
	Others (specify)	1	0	1	0
156.	If other cause of death, please specify	……………………………<br>…………………….			
157.	Day of discharge from hospital (BS)				
